# Supplementary material for: Proliferating coacervate droplets as the missing link between chemistry and biology in the origins of life
Source: Nat Commun. 2021 Sep 24;12:5487. doi: 10.1038/s41467-021-25530-6 (PMC8463549; doi:10.1038/s41467-021-25530-6)
Supplement: Supplementary file 1 — Supplementary Information [file 41467_2021_25530_MOESM1_ESM.pdf]

Supplementary Information

Proliferating coacervate droplets as the missing link between chemistry and biology in the origins of life

Muneyuki Matsuo & Kensuke Kurihara

INDEX

Supplementary Methods

Chemicals and apparatus

General preparation and purification of oligomer

Measurement of turbidity

Recursive growth and division of LLPS droplets

Confocal laser scanning microscopy observations of RNA, DNA, and/or lipid incorporation into droplets

Flow cytometry analysis of droplets incorporating RNA, DNA, and/or lipid

Raman spectroscopy analysis of droplets and droplets with/without DNA and/or phospholipids

Spectrofluorometric measurement of phase transfer of RNA, DNA, or lipid

Supplementary Figures

1. Scheme of synthesis of monomer precursor $\mathbf{M_{pre}}$ and thioesterified glycine (Gly-SBn·HCl)
2. ^{1}H NMR spectrum of $(Boc\text{-}Cys\text{-}SBn)_2$ with CD_3OD
3. ^{1}H NMR spectra of $\mathbf{M_{pre}}$ with CD_3OD and D_2O
4. DIC microscopy images of solutions after addition of either $\mathbf{M_{pre}}$ or cystine dihydrochloride, BnSH, and DTT (25 mM) or cystine dihydrochloride to deionised water.
5. ^{1}H NMR spectra of Boc-Gly-SBn with $CDCl_3$ and Gly-SBn·HCl with CD_3OD
6. Photographs of solutions after addition of $\mathbf{M_{pre}}$ and DTT or Gly-SBn and DTT

7. Photographs of solutions after addition of $\mathbf{M_{pre}}$ and DTT under various aqueous conditions

8. ^{1}H NMR spectrum of CD_3CN phase used for purification of reaction products

9. ^{1}H NMR spectrum of purified product with DMSO-d_6 and comparison of the spectra of $\mathbf{M_{pre}}$ and oligopeptides

10. ESI-TOF mass spectra and analysis of reaction products

11. DIC microscopy images of solutions containing dissolved oligopeptides and BnSH with/without DTT

12. Composition ratios of peptides and benzyl mercaptan in formed droplets

13. Monitoring of droplet formation reaction by fluorescence spectroscopies and ^{1}H NMR

14. Monitoring of progress of autocatalytic reaction with ^{1}H NMR

15. Comparison of particle size distribution before/after extrusion

16. Raw image of Figure 5b

17. Monitoring of LLPS droplet size during the growth-division process induced by repeated nutrient addition and stimulus cycles

18. Relative number of droplets after each generation

19. Time-course changes in droplet size and reaction rate

20. Control experiment for growth of droplet

21. CLS fluorescence microscopy images of the dispersion of LLPS droplets after addition of the fluorescent probe tagged–nucleic acid or lipid solutions

22. Raman microspectrometry of an LLPS droplet

23. FACS analysis of particle size distribution after addition of BODIPY-HPC and/or TAMRA-RNA

24. Raman microspectroscopic images of an LLPS droplet after incorporation of RNA or DNA and/or lipids

25. Fluorescence spectroscopic analysis of the concentrations of oligopeptides in biopolymers

Supplementary Table

1. Summary of Raman microspectroscopic analysis of an LLPS droplet with/without RNA or DNA and/or lipids

Supplementary Note

1. Plausible prebiotic synthesis of components
2. Separation of droplets into water phase and oil phase by centrifugation.

Supplementary Movie

1. Droplet formation
2. Droplet fusion

Supplementary References

Supplementary Methods

Chemicals and apparatus. L-cysteine hydrochloride monohydrate, L-cystine dihydrochloride, *N*,*N*′-bis(*tert*-butoxycarbonyl)-L-cystine [(Boc-Cys-OH)$_2$], benzyl mercaptan (BnSH), dithiothreitol (DTT), fluorescamine, and 1,2-dipalmitoyl-*sn*-glycero-3-phosphocholine (DPPC) were purchased from Tokyo Chemical Industry Co., Ltd. (Tokyo, Japan). *N*,*N*′-dicyclohexylcarbodiimide, 1-hydroxybenzotriazole, *N*,*N*-diisopropylethylamine, hexane, ethyl acetate, super-dehydrated dimethyl sulfoxide, and rhodamine-labelled lipid Texas Red-tagged DHPE (*N*-(Texas Red sulfonyl)-1,2-dihexadecanoyl-*sn*-glycero-3-phosphoethanolamine, triethylammonium salt) (Texas Red-DHPE) were purchased from Fujifilm Wako Pure Chemical Corp. (Osaka, Japan). Hydrogen chloride in ethyl acetate (4N HCl/ethyl acetate) was purchased from Kokusan Chemical Co., Ltd. (Kanagawa, Japan). Tenfold concentrated

phosphate buffer solution (pH 7.4, 67 mM after dilution) was purchased from Kanto Chemical Co., Inc. (Tokyo, Japan). BODIPY-tagged phospholipid 2-(4,4-difluoro-5,7-dimethyl-4-bora-3a,4a-diaza-*s*-indacene-3-dodecanoyl)-1-hexadecanoyl-*sn*-glycero-3-phosphocholine (BODIPY-HPC) was obtained from Thermo Fisher Scientific Inc. (Tokyo, Japan). Synthesised fluorescent-labelled single-stranded RNAs (5′-FITC-GACAGCAUCGCCAGUCACUA-3′ and 5′-TAMRA-GACAGCAUCGCCAGUCACUA-3′) and DNAs (5′-FITC-GACAGCATCGCCAGTCACTA-3′ and 5′-TAMRA-GACAGCATCGCCAGTCACTA-3′) were obtained from FASMAC (Kanagawa, Japan). RNase-free water (Water for Molecular Biology) was purchased from Merck Japan (Tokyo, Japan) and used as the water in all of the RNA experiments and control experiments. Tuning solution for mass spectrometry (Standards Chemical Kit PN4456735, part number: 4457953, MW 829.0) was purchased from AB Sciex Pte., Ltd. (Framingham, MA, U.S.A.).

^{1}H NMR and ^{13}C NMR spectra were recorded using a spectrometer (JNM-ECS400, JEOL, Tokyo, Japan). Chemical reactions were monitored with a spectrometer (JNM-ECM600, JEOL). Flash column chromatography was performed using silica gel columns (RediSep® Normal-phase Silica Flash Columns, 20–40 μm mesh, pore size 60 Å, Teledyne Isco, Inc., Lincoln, NE, USA) on a medium-pressure liquid chromatography purification system (CombiFlash Rf 150, Teledyne Isco, Inc., Lincoln, NE, USA). The crude product was purified using a preparative high-performance liquid chromatography system (LC-Forte/R, YMC Co., Kyoto, Japan). Electrospray ionisation time-of-flight mass spectrometry measurements were carried out using a mass spectrometer (TripleTOF 5600, AB Sciex Pte. Ltd.). The turbidity of the solution was measured with a microvolume UV-Vis spectrophotometer (Nano Drop 2000c, Thermo Fisher Scientific, MA, USA). The hydrodynamic diameters of the molecular assemblies in the solution were measured with a dynamic light-scattering particle-size analyser (ELSZ-1000, Otsuka Electronics Co., Ltd., Osaka, Japan). Microscopic observations were made with an inverted microscope (Eclipse Ti-E, Nikon Instruments Inc., Tokyo, Japan) equipped with a confocal laser scanner unit (CSU-W1, Yokogawa Electric Corp., Tokyo, Japan)

and a sCMOS camera unit (Zyla 4.2 plus, Andor Technology Ltd., Belfast, United Kingdom) with 1024 × 1024 active pixels (λ_{ex} = 488 nm, λ_{em} = 500–550 nm). The microscopic images were recorded using an imaging software (NIS-Elements AR ver. 5.11.01, Nikon Instruments Inc.). A spectrofluorometer (SPEX Fluorolog 3-21, Horiba, Kyoto, Japan) was used for photon counting. Raman images and spectra were obtained using a laser Raman microscope system (RAMAN-11, Nanophoton Corp., Osaka, Japan) equipped with an upright metallurgical microscope (Eclipse LV150, Nikon Co., Tokyo, Japan) with a 600 g/mm grating and neutral-density filter (255, transmittance: 99%). The laser beam was focused on a sample with an objective lens (60×/1.0 NA water immersion). The central wavenumbers were 2000 cm^{-1} for wavenumber ranges of 700–3000 cm^{-1}, and 2800 cm^{-1} for wavenumber ranges of 1500–3500 cm^{-1}. The droplet dispersion was placed in a chamber on a slide glass, and a cover glass was put on the dispersion. Raman spectra from each of four points in the core and shell of the droplets were measured to obtain a single Raman image. The irradiated laser intensity and exposure time at each point were 0.57–0.59 mW (532 nm) and 15 s, respectively.

General preparation and purification of oligomer. $\mathbf{M_{pre}}$ [$(Cys\text{-}SBn)_2 \cdot 2HCl$] was synthesised as shown in Supplementary Figure 1. $\mathbf{M_{pre}}$ (11 mg, 20 mmol) and reductant dithiothreitol (DTT, 8 mg, 50 mmol) were added to the deionised water (2 mL) and shaken for 15 s. $\mathbf{M_{pre}}$ was reduced to **M** (Cys-SBn·HCl) to initiate polymerisation. At 24 h after mixing $\mathbf{M_{pre}}$ and DTT, the solution (500 μL) was lyophilised to remove both water and BnSH. The resultant powder was washed with 500 μL of acetonitrile five times to remove DTT and oxidised DTT; the precipitate was then obtained as a white powder.

Measurement of turbidity. $\mathbf{M_{pre}}$ (5 mg, 10 mmol) and/or the reductant DTT (4 mg, 25 mmol) were added to 1 mL of deionised water and shaken for 15 s. Two microliters of aqueous solution were removed from the solution and assayed. The same procedure was repeated for 24 h. Absorbances at

400 nm were measured with a microvolume UV-Vis spectrophotometer (Nano Drop 2000c) and were equated to the turbidities. Turbidity measurements of Gly-SBn·HCl and DTT were performed in the presence of $\mathbf{M_{pre}}$ as described above.

Recursive growth and division of LLPS droplets. $\mathbf{M_{pre}}$ (11 mg, 20 mmol) and DTT (8 mg, 50 mmol) were added to deionised water (2 mL) and vortexed at room temperature for 15 s. One hundred microliters of the solution were poured into a quartz cuvette. The particle size distribution of first-generation LLPS droplets was monitored with a particle size analyser at room temperature every 10 min for 24 h. The remaining dispersion was incubated for 24 h. To analyse the particle size of second-generation LLPS droplets, the dispersion was extruded through a Nuclepore® polycarbonate filter (pore size 100 nm, Whatman) five times 24 h after mixing. Immediately after extrusion, deionised water was added to make the concentrations of $\mathbf{M_{pre}}$ and DTT in the dispersion the same as the initial concentrations, and the same amounts of $\mathbf{M_{pre}}$ and DTT were added again for self-reproduction of LLPS droplets. The particle size change was monitored with the particle analyser at room temperature for 24 h immediately after vortex mixing. This series of processes was repeated six times.

Confocal laser scanning microscopy observations of RNA, DNA, and/or lipid incorporation into droplets. An LLPS droplet dispersion was prepared by mixing $\mathbf{M_{pre}}$ and DTT and incubating the mixture for 24 h. A 13-μL droplet of the dispersion and 13 μL of TAMRA or FITC-tagged single strand RNA (ssRNA) or single-strand DNA (ssDNA) solution (1 μM) were placed on a glass plate and then mixed. The plate was then immediately covered with a cover glass using a spacer. The specimen was observed with a confocal laser scanning fluorescence microscope. Similarly, either (1) 13 μL of the LLPS droplet dispersion and 13 μL of Texas Red-DHPE or BODIPY-HPC solution (1 μM) or (2) 9 μL of the LLPS droplet dispersion, 9 μL of ssRNA solution, and 9 μL of lipid solution were mixed and obtained as a sample.

Flow cytometry analysis of droplets incorporating RNA, DNA, and/or lipid. Three kinds of biomolecular solutions (TAMRA-RNA aq., 1 μM, 500 μL; BODIPY-HPC aq., 1 μM, 500 μL; an equivolume mixture of TAMRA-RNA aq. and BODIPY-HPC aq., 0.5 μM each, 1000 μL) were prepared. A sample solution was prepared by mixing each of the above biomolecular solutions and 500 μL of an LLPS droplet dispersion consisting of $\mathbf{M_{pre}}$ (10 mM) and DTT (25 mM). The mixture at 5 min and 24 h after mixing was transferred to a fluorescence-activated cell sorter (FACS) using the Cell Sorter SH800 Software ver. 3.0.0. The intensities of scattered light and fluorescence were detected by the FACS on a mass scale (counting 10,000 droplets, TAMRA, λ_{ex} = 561 nm, λ_{em} = 600 ± 30 nm; FITC, λ_{ex} = 488 nm, λ_{em} = 525 ± 25 nm; Texas Red, λ_{ex} = 561 nm, λ_{em} = 600 ± 30 nm; BODIPY, λ_{ex} 488 nm, λ_{em} = 525 ± 25 nm). The FACS operation and analysis procedures were based on a previous study[1]. The probability density of each population was calculated with a kernel density estimation method.

Raman spectroscopy analysis of droplets and droplets with/without DNA and/or phospholipids. In order to investigate the ratio of hydrophobic and hydrophilic components in both the central and peripheral parts of the LLPS droplets, the Raman spectra of each part were measured by laser Raman microscopy. $\mathbf{M_{pre}}$ (2.5 mg, 4.5 mmol) and DTT (2 mg, 12.5 mmol) were mixed, and then 100 μL of deionised water was added immediately. The mixture was stirred by vortexing for 15 s. One hour after this stirring, the droplet dispersion (25 μL) was observed with a laser Raman microscope. Raman imaging was performed at 3050 cm^{-1} to optimise the imaging. The benzene ring–derived peak of benzyl mercaptan (~1000 cm^{-1}) as the hydrophobic component and the water-derived peak (~3400 cm^{-1}) as the hydrophilic component were detected, and the benzene ring/water ratio (B/W) was compared between the central part (defined as C[B/W]) and the peripheral part (defined as P[B/W]). In the case of the droplet containing DNA, 100-μL solutions of DNA (1 μM, 20-mer) were mixed with

the 100-μL droplet dispersion for 1-2 h after mixing $\mathbf{M_{pre}}$ and DTT. We prepared a specimen: 25-μL aliquots of the droplet dispersion dispensed from the mixed dispersion. In the case of the droplet containing phospholipids, 100-μL aliquots of DPPC solutions (1 μM) were mixed with 100 μL of the droplet dispersion for 1–2 h after mixing $\mathbf{M_{pre}}$ and DTT. A 25-μL aliquot of the droplet dispersion was dispensed and examined. In the case of the droplet containing DNA and phospholipids, 100-μL aliquots of each of the DNA (1 μM) and DPPC (1 μM) solutions were mixed with a 100-μL aliquot of droplet dispersion for 1–2 h after mixing the $\mathbf{M_{pre}}$ and DTT. A 25-μL aliquot of the droplet dispersion was dispensed from the mixed dispersion. Due to the property of our Raman spectorometer, the measurements were made separately for low and high wavenumbers, and then the spectrum baselines were aligned and combined.

Spectrofluorometric measurement of phase transfer of RNA, DNA, or lipid. Four kinds of biomolecule-containing solutions (TAMRA-RNA aq., FITC-RNA aq., Texas Red–DHPE aq., or BODIPY-HPC aq.; 1 μM each) were prepared. An aqueous oligopeptide solution (with oligopeptides or without oligopeptides) was prepared by redispersing the extracted oligopeptides in the RNase-free water according to the above protocols (See **General preparation and purification of oligomer**). A biomolecule-containing solution (100 μL) and an aqueous oligopeptide solution (100 μL) were mixed and then incubated for 24 h on a 200-μL layer of BnSH solution. After incubation, a 100-μL aliquot of the aqueous solution was dispensed into a cuvette, and the fluorescence spectrum was measured by counting photons with a spectrofluorometer. The measurement wavelengths of each fluorescent molecule were as follows: TAMRA, λ_{ex} = 525 nm, λ_{em} = 535–800 nm; FITC, λ_{ex} = 485 nm, λ_{em} = 500–800 nm; Texas Red, λ_{ex} = 565 nm, λ_{em} = 575–800 nm; BODIPY, λ_{ex} 485 nm, λ_{em} = 500–800 nm).

Supplementary Figures

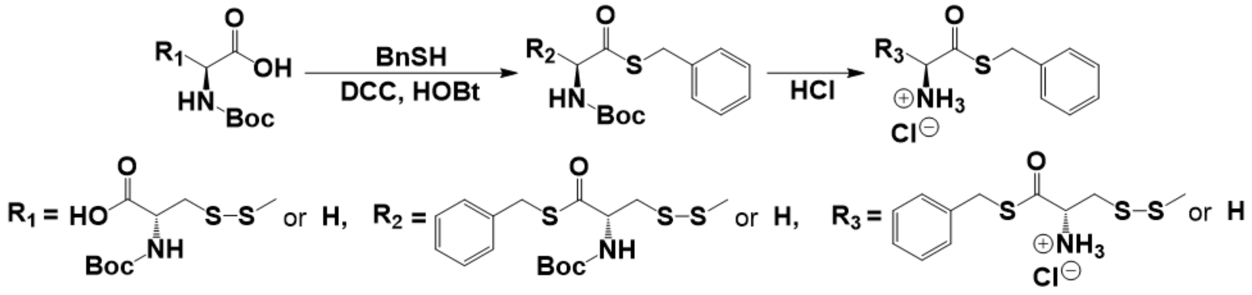

Fig. 1 Scheme of synthesis of monomer precursor $\mathbf{M}_{\mathbf{pre}}$ and thioesterified glycine (Gly-SBn·HCl). The actual synthesis process is described in the Methods section of the article.

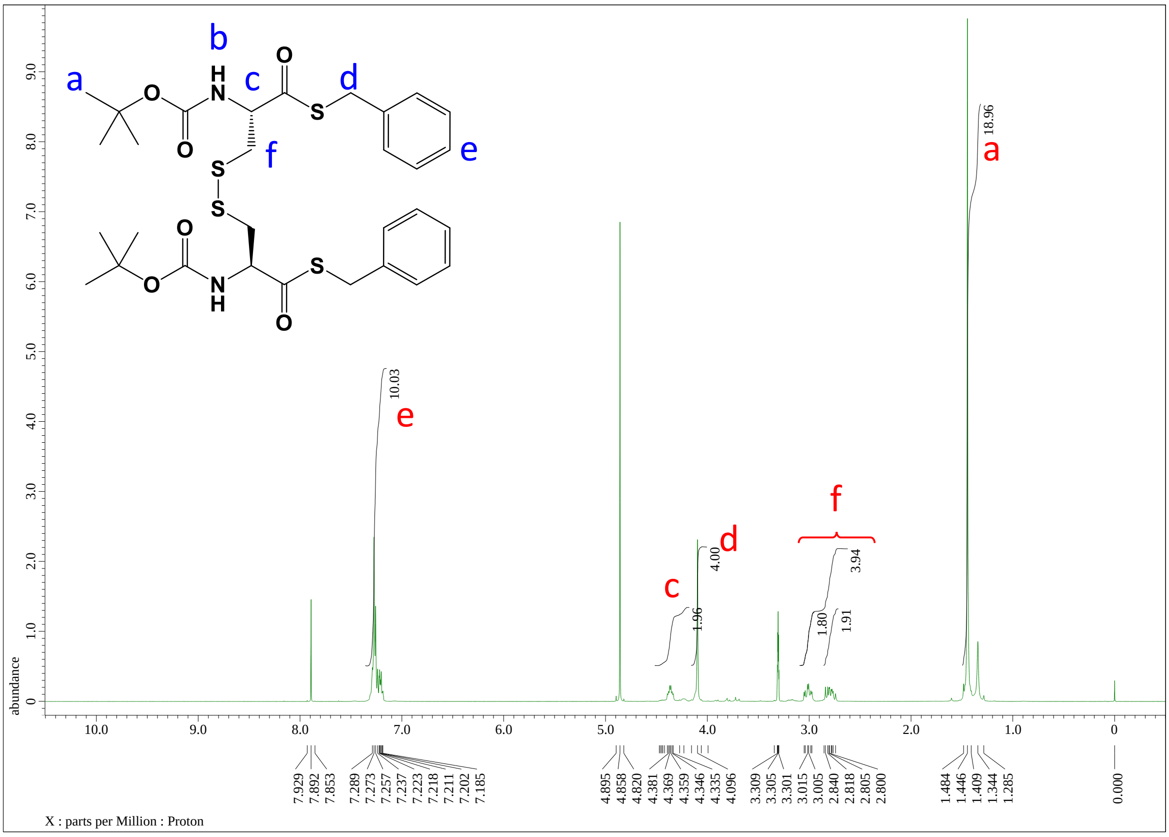

Fig. 2 ^{1}H NMR spectrum of (Boc-Cys-SBn)$_2$ with CD_3OD. The same lowercase letters in the structural formula (blue) and spectrum (red) represent protons and their corresponding peaks. The synthesis process is described in the Methods section of the article.

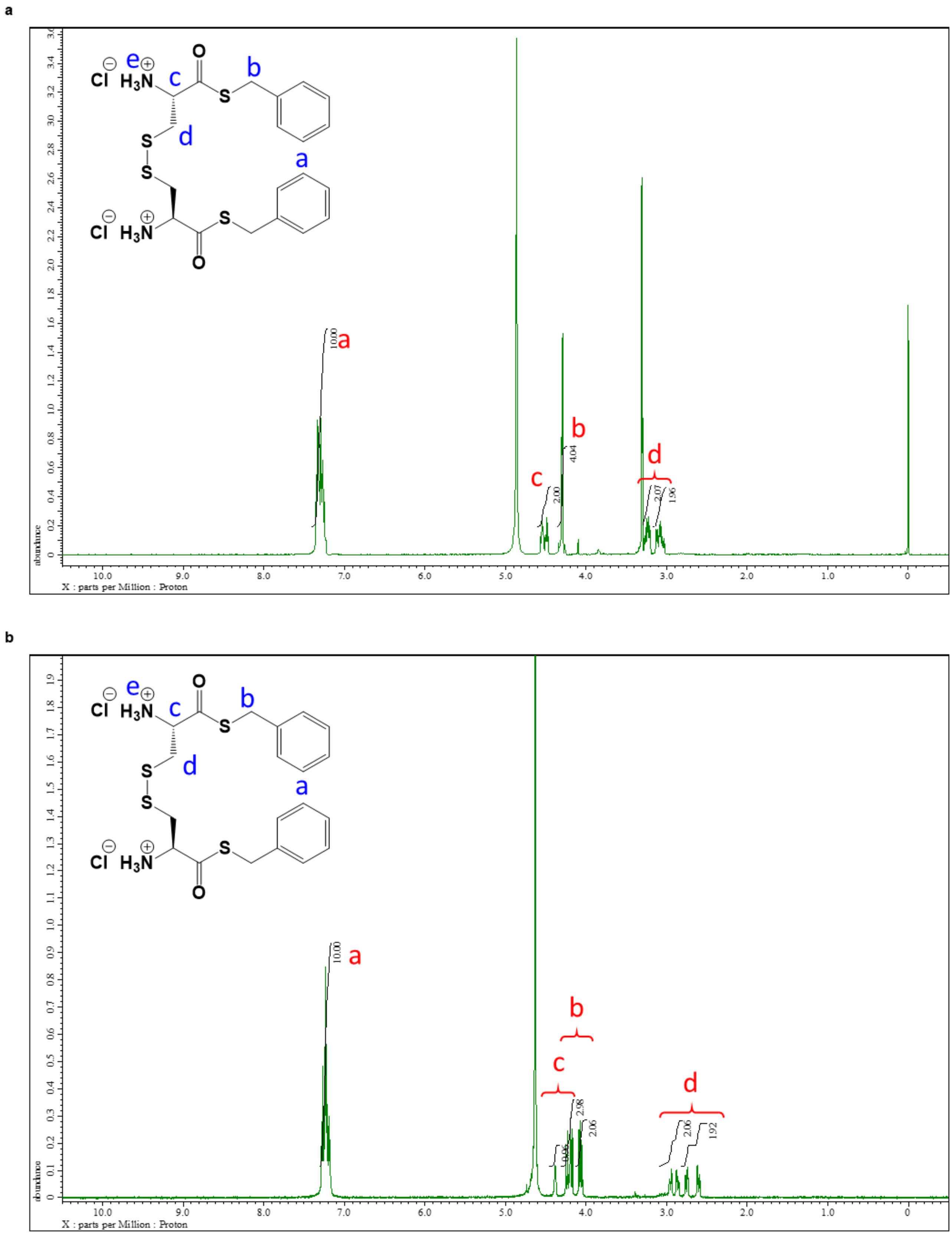

Fig. 3 ^{1}H NMR spectra of $\mathbf{M_{pre}}$ with **a** CD_3OD or **b** D_2O. The same lowercase letters in the structural formula (blue) and spectrum (red) represent protons and their corresponding peaks. The synthesis

process is described in the Methods section of the article.

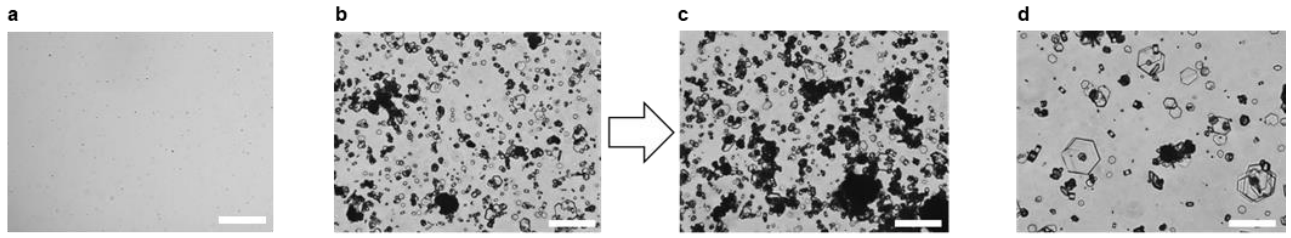

Fig. 4 DIC microscopy images of solutions after addition of **a** 10 mM $\mathbf{M_{pre}}$, **b** after addition of cystine dihydrochloride (10 mM), BnSH (20 mM), and DTT (25 mM) at 5 min and **c** at 12 h, and **d** after addition of cystine dihydrochloride (10 mM) to deionised water. Scale bars represent 50 µm. The trends were verified by more than three trials.

a

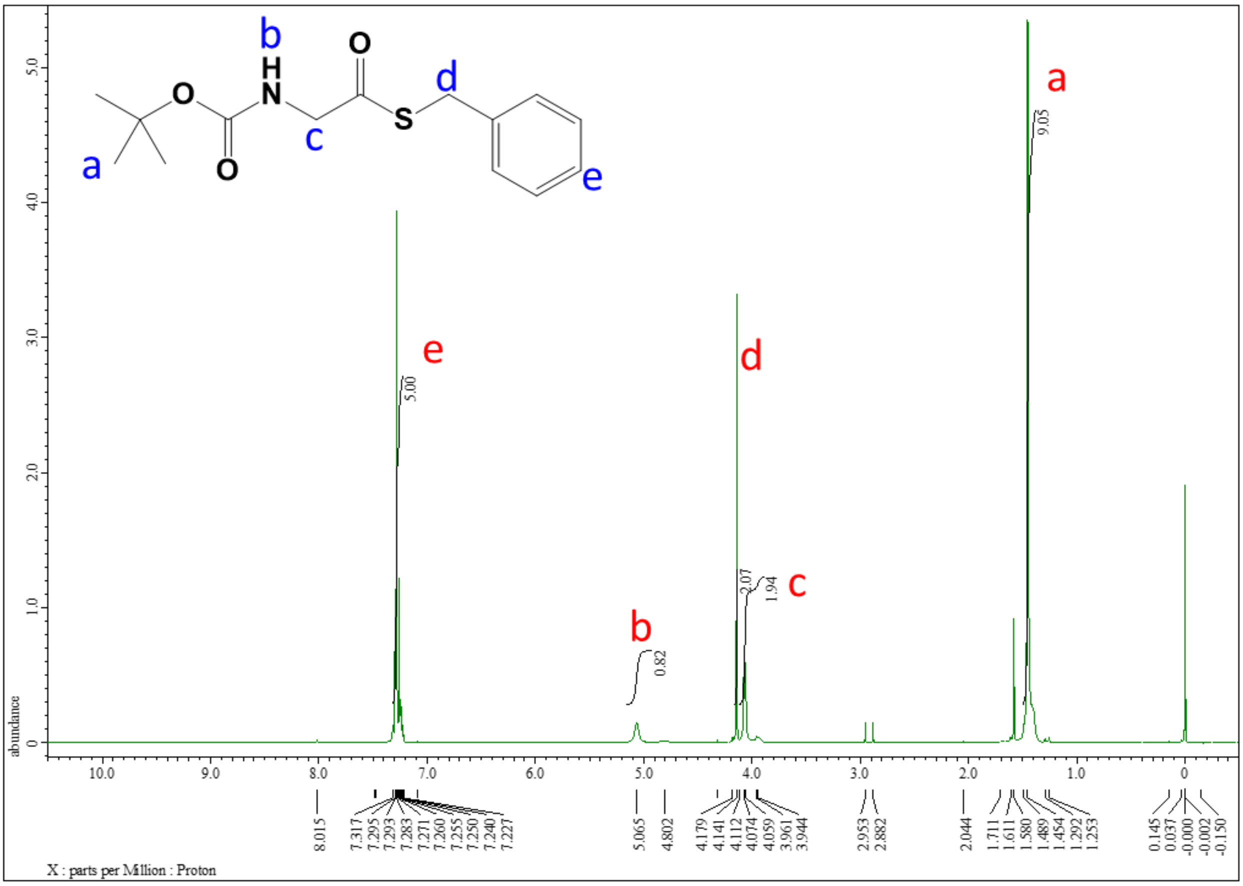

b

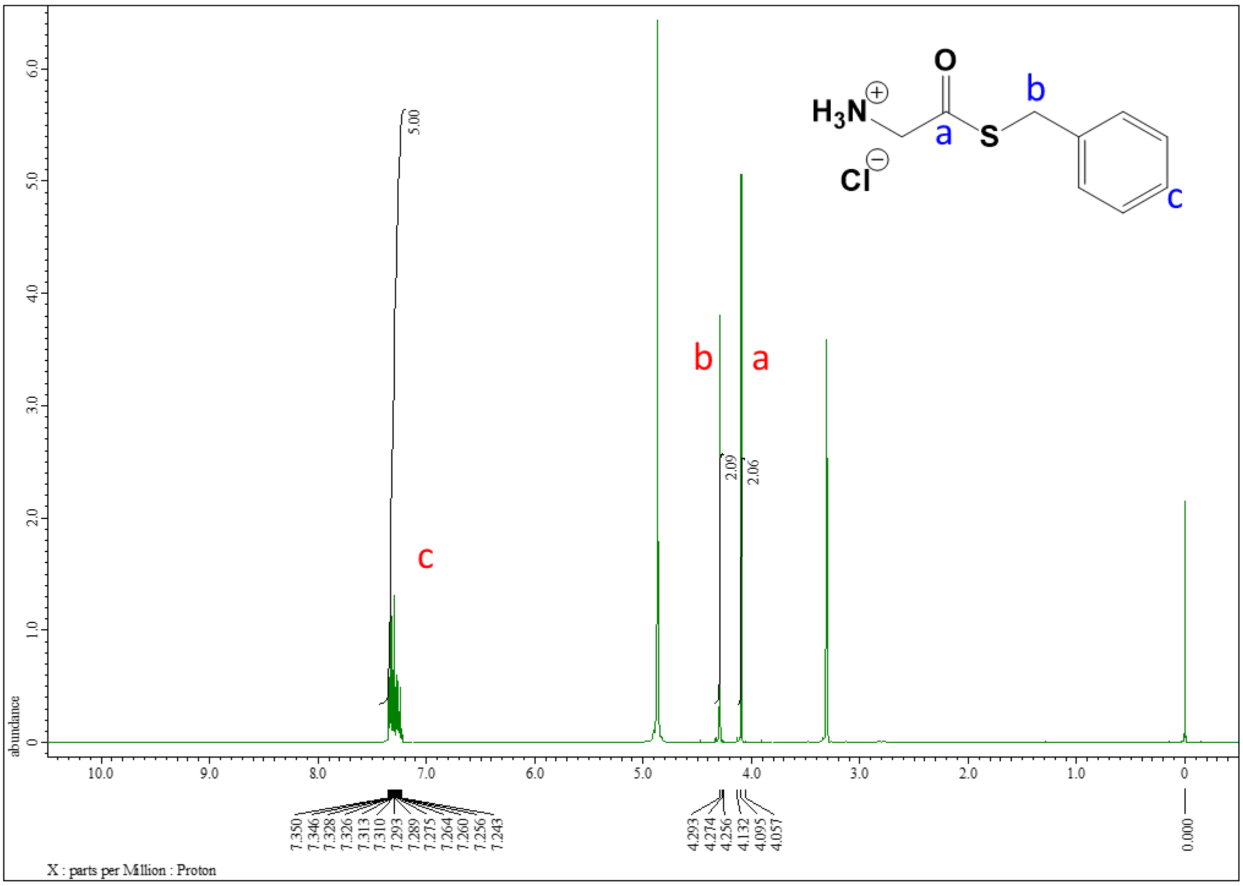

Fig. 5 ^{1}H NMR spectra of **a** Boc-Gly-SBn with $CDCl_3$ and **b** Gly-SBn·HCl with CD_3OD. The same lowercase letters in the structural formula (blue) and spectrum (red) represent protons and their corresponding peaks. The synthesis process is described in the Methods section of the article.

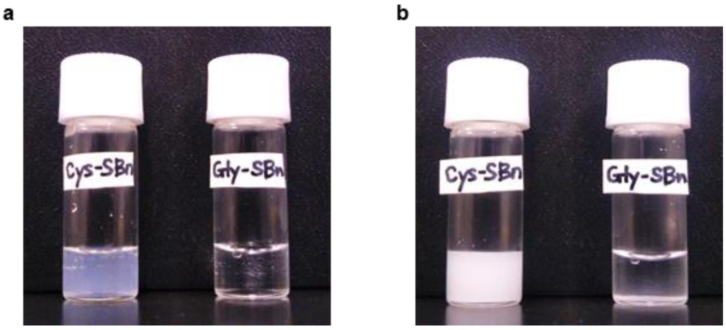

Fig. 6 Photographs of solutions at **a** 5 min and **b** 24 h after addition of $\mathbf{M}_{\mathbf{pre}}$ (10 mM) and DTT (25 mM) (left vial) or Gly-SBn·HCl (20 mM) and DTT (25 mM) (right vial).

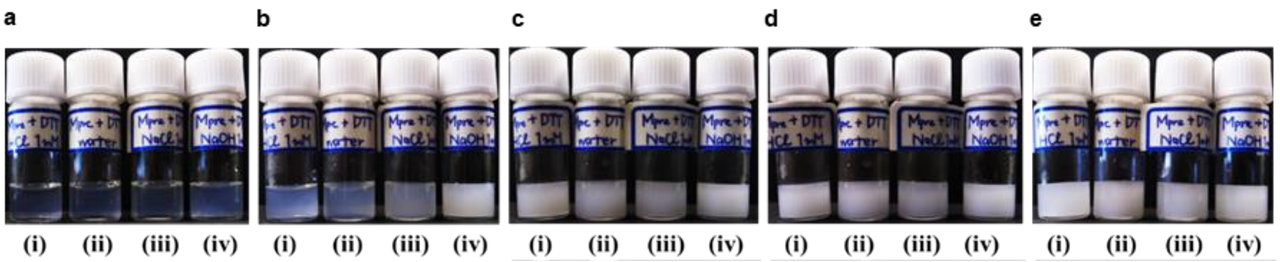

Fig. 7 Photographs of the solution of $\mathbf{M_{pre}}$ (10 mM) and DTT (25 mM) at various pHs and ion concentrations **a** 5 min after mixing, **b** 30 min after mixing, **c** 1.5 h after mixing, **d** 3 h after mixing, and **e** 12 h after mixing. Each aqueous solution was prepared containing (i) hydrochloric acid (1 mM, pH = 3), (ii) deionised water (pH = 7), (iii) sodium chloride (1 mM, pH = 7), and (iv) sodium hydroxide (1 mM, pH = 11).

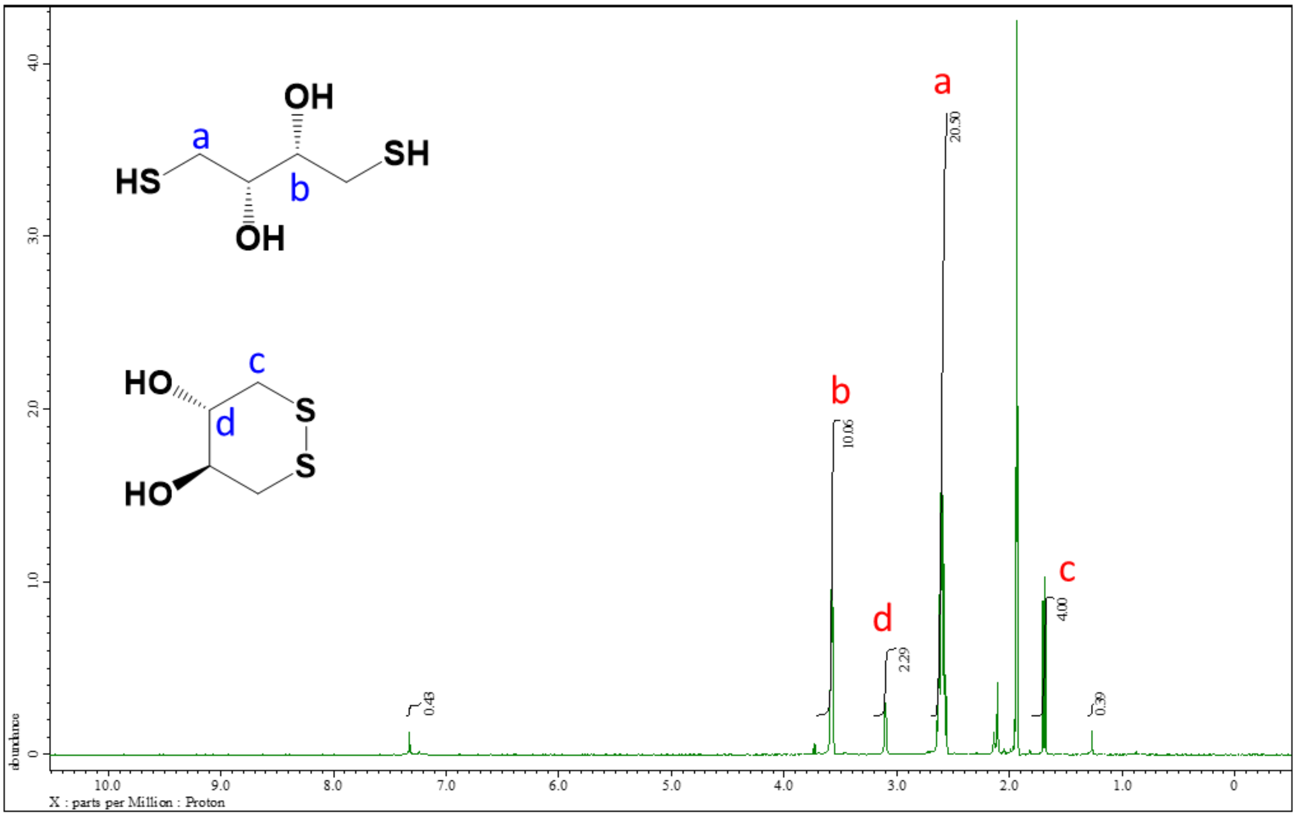

Fig. 8 ^{1}H NMR spectrum of CD_3CN phase used for purification of reaction products. The eluates were identified as DTT and oxidised DTT. The same lowercase letters in the structural formula (blue) and spectrum (red) represent protons and their corresponding peaks.

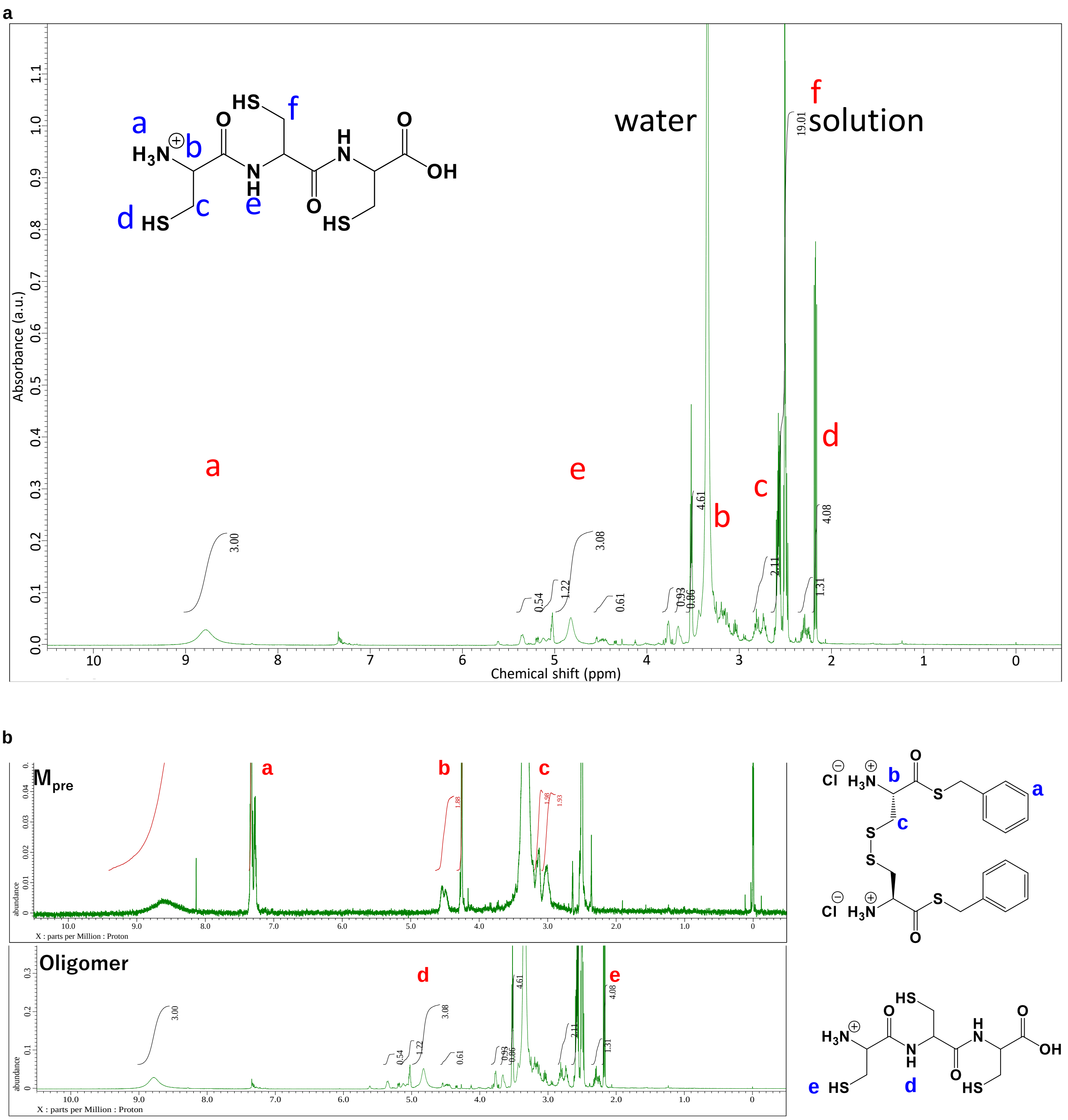

Fig. 9 ^{1}H NMR spectrum of purified product. **a** ^{1}H NMR spectrum of the purified reaction products with DMSO-d_6, which were identified as oligopeptides. **b** Comparison of ^{1}H NMR spectra of $\mathbf{M}_{\mathbf{pre}}$ and oligopeptides. The peak of the benzene ring (peak a in Supplementary Figure 9b) and the peaks near the disulfide (b, c) almost disappeared, whereas the amide proton (d) and thiol proton (e) were newly detected. The same lowercase letters in the structural formula (blue) and spectrum (red) represent protons and their corresponding peaks.

a

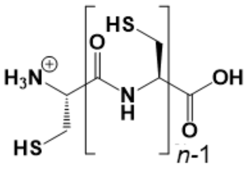

Non-thioesterified product (n**A**)

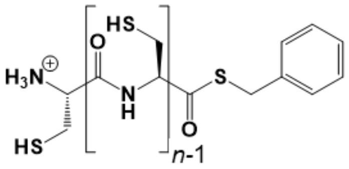

Thioesterified product (n**B**)

Product group		Number of units			
		1	2	3	4
Non-thioesterified (**A**)	found	122.034	225.0723	***328.0726***	431.0852
	calcd.	(122.027)	(225.0362)	(328.0454)	(431.0546)
Thioesterified (**B**)	found	**228.0634**	**331.0784**	434.0830	–
	calcd.	(228.0511)	(331.0603)	(434.0695)	(537.0787)

b

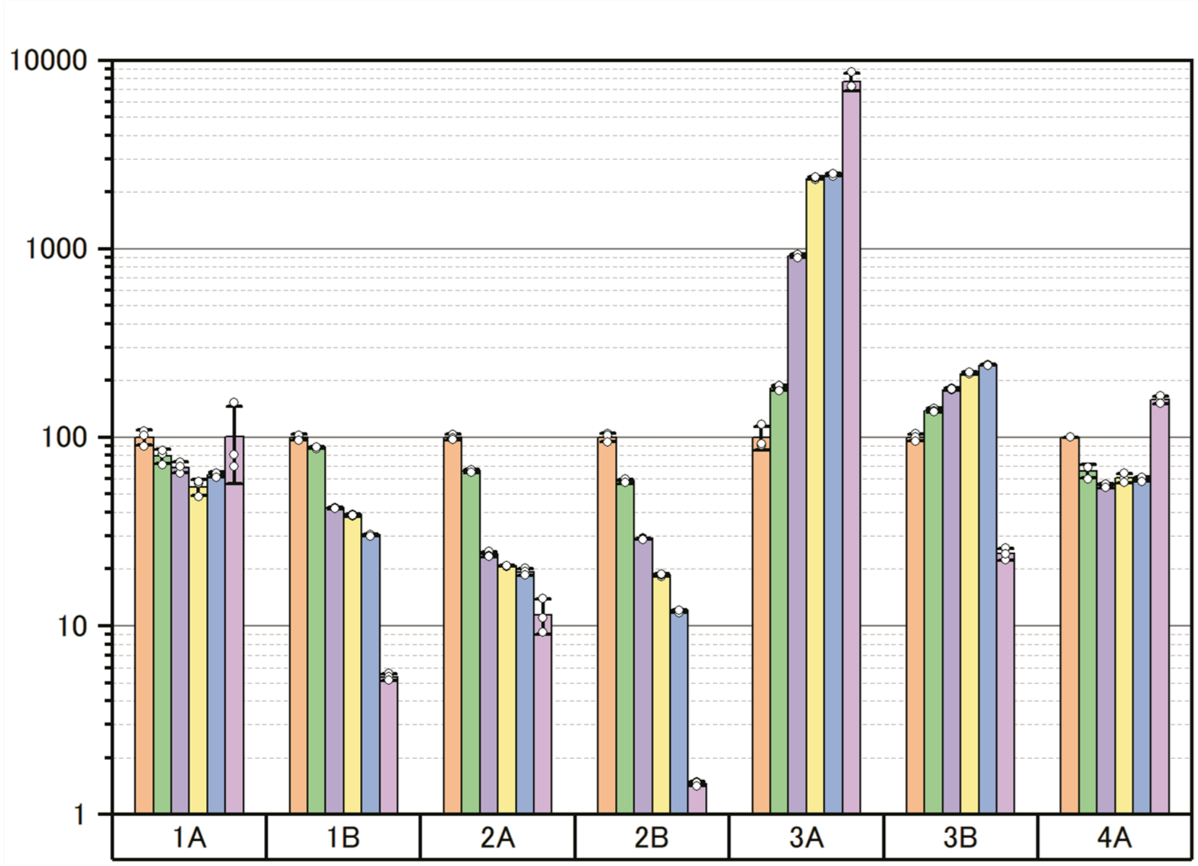

c

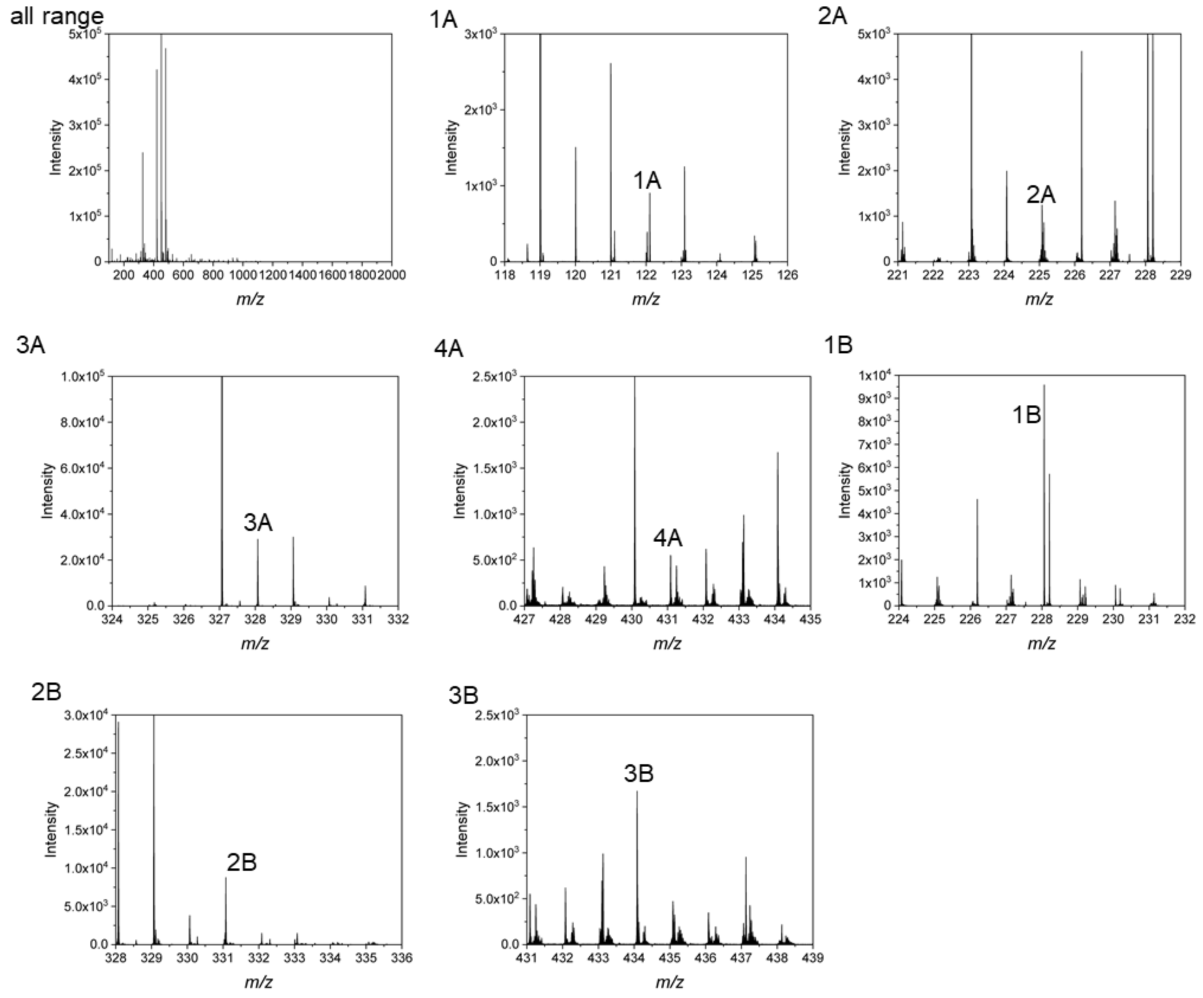

Fig. 10 ESI-TOF mass spectra and analysis of reaction products. **a** The upper scheme indicates the molecular structures of the non-thioesterified (left) and thioesterified (right) product. Here, *n* is polymerisation degree (an integer ≥1, and $n - 1$ is the number of units). The lower table shows the detected mass-to-charge ratio (*m*/*z*) values in the ESI-TOF mass spectrum. For the products 3 h after mixing $\mathbf{M_{pre}}$ and DTT, the *m*/*z* values of the monomers, dipeptides, tripeptides, tetrapeptides, pentapeptides, and hexapeptides of non-thioesterified and thioesterified products were assigned by focusing on only the *m*/*z* values with more than 200 intensity units in the ESI-TOF mass spectrum. In the table, bold numbers indicate intensity values of 2000 or more, italic numbers indicate intensity values of 20,000 or more, and - indicates no detection. **b** Intensity quantified by ESI-TOF mass spectrometry versus time of non-thioesterified peptides (**A**s) and thioesterified peptides (**B**s) in the

dispersion. Bar graphs from left to right in each peptide (1**A**, 2**A**…) are the intensities 0 min, 30 min, 60 min, 120 min, 180 min, and 24 h after mixing $\mathbf{M_{pre}}$ and DTT. Data are presented as mean values +/- SD. The vertical axis is the normalised intensity of each peptide (left to right, 1**A**, 1**B**, ...) detected in the sample at 0 min, where the initial intensity was set to 100. Smaller degrees of polymerisation (n = 1, 2) decreased with time, whereas larger degrees of polymerisation (n = 3, 4) increased at 24 h after mixing $\mathbf{M_{pre}}$ and DTT. These results suggest that the peptide monomers were gradually polymerised to form oligopeptides. Corresponding data were overlayed as dot plots. **c** All *m*/*z* values obtained from the ESI-TOF mass spectrum are shown. In the spectra, *n***A**, *n***B** (n = 1, 2 …) means detected peptides, where **A** means non-thioesterified ester, **B** means thioesterified ester, and *n* means degrees of polymerisation. Source Data of 10b and 10c is provided in the Source Data File.

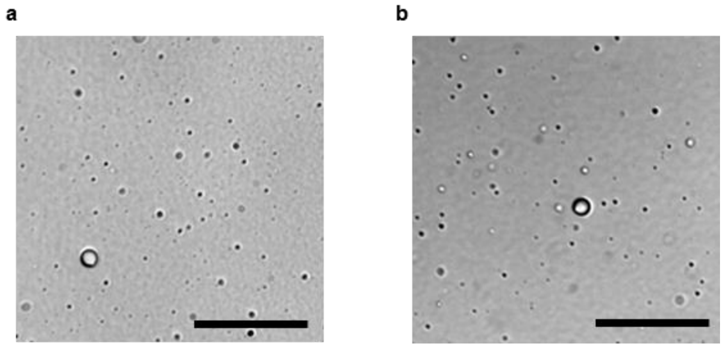

Fig. 11 DIC microscopy images of solutions. **a** Mixtures of oligopeptides (10 mM) and BnSH (10 mM). **b** Mixtures of oligopeptides (10 mM), BnSH (10 mM) and DTT (25 mM). The concentrations of these molecules were calculated based on conversion from $\mathbf{M_{pre}}$. The fact that both solutions formed droplets indicated that both the oligopeptides and BnSH were required for droplet formation. The results were verified by five trials. Scale bars represent 20 μm.

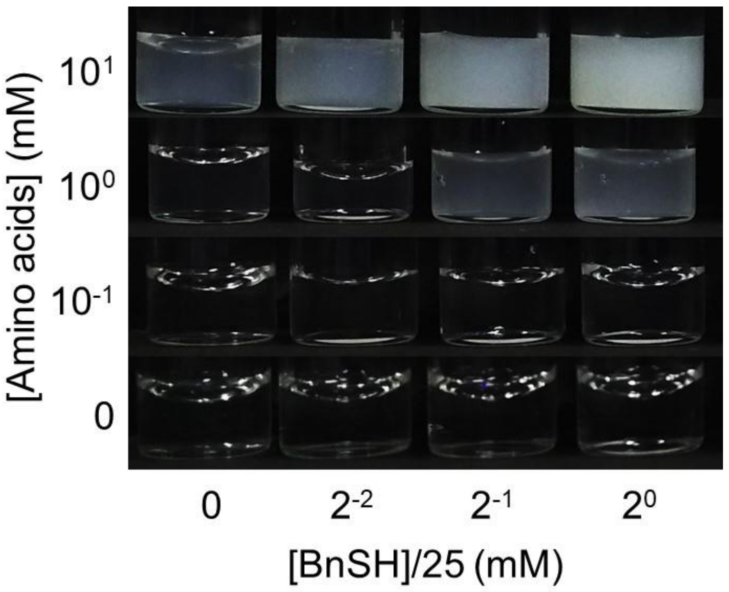

Fig. 12 Composition ratios of peptides and benzyl mercaptan in formed droplets. **a** Photographs showing the dependence of droplet formation on the concentration ratios of benzyl mercaptan (BnSH) to peptides. The concentrations of the oligopeptides are expressed in terms of amino acid concentrations. Each specimen was prepared by adding BnSH and deionized water to the peptides obtained by the same procedure shown as Figure 3a.

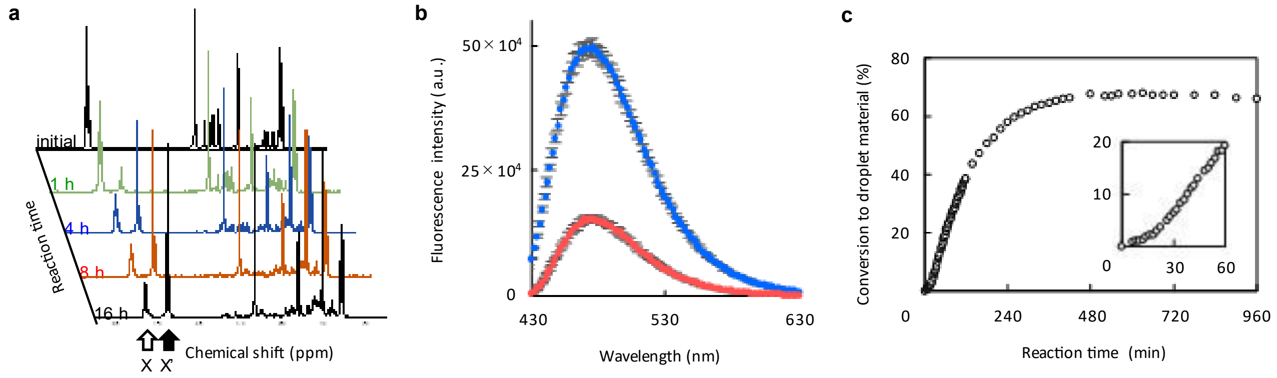

Fig. 13 Monitoring of the process of droplet formation by f ^{1}H NMR and fluorescence spectroscopy. **a** Temporal evolution of ^{1}H NMR spectra of aqueous solution of $\mathbf{M_{pre}}$ (10 mM) and DTT (25 mM), monitored for 16 h after mixing. Peaks X and X' were assigned to the benzyl moieties before and after BnSH was removed, respectively. The conversion to droplet material (%) was estimated from the integrated peak areas and equated to 100 × X' / (X + X'), where X and X' are the integrated peak areas

of the benzyl moieties before and after BnSH was removed, respectively. The ranges of the chemical shifts of the X and X' peaks were 7.0–7.5 and 6.5–7.0, respectively. **b** Fluorescence spectrum of fluorescamine in the solution 24 h after mixing with 10 mM $\mathbf{M_{pre}}$ and 25 mM DTT (red) as well as in a solution of cysteine hydrochloride (20 mM, blue). The $\mathbf{M_{pre}}$ residual percentage was calculated from the fluorescence intensity of the solution relative to that measured immediately after mixing $\mathbf{M_{pre}}$ and DTT, which was designated as 100%. **c** A plot of the conversion to droplet material (%) from 0 h to 16 h after addition. The inset shows a magnified view of Supplementary Figure 13c up to 60 min. Source Data of 13b and 13c is provided in the Source Data File.

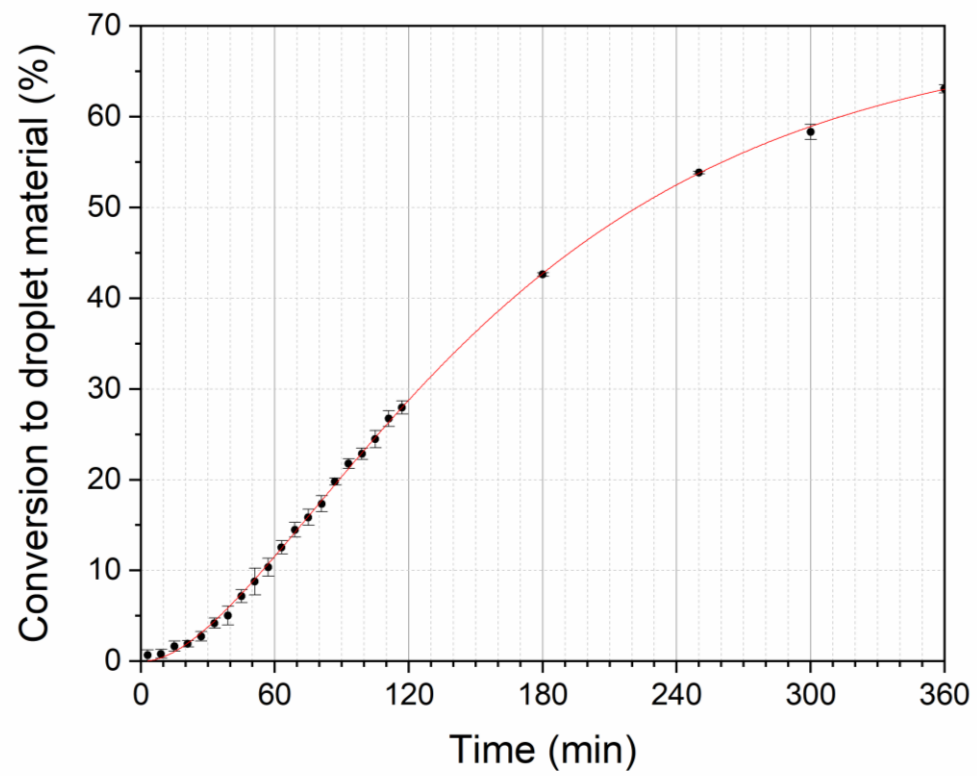

Iterative algorithm	Levenberg-Marquardt Method
Formula	y = a*(1 + (d-1)*exp(-k*(x-xc)))^(1/(1-d))
a	69.70121±0.66271
xc	77.36148±1.94612
d	0.46532±0.0266
k	0.00822±2.50981E-4
Reduced Chi-Sqr	0.40012
R-Square (COD)	0.99987
Adj. R-Square	0.99985

Fig. 14 Conversion to droplet material (%) from NMR measurements and the equation for a sigmoidal

curve. After adding 5 mg of $\mathbf{M_{pre}}$ and 4 mg of DTT to 1 mL of D_2O, the change in droplet formation percentage was examined with ^{1}H NMR. The droplet formation percentage was calculated from the integrated area (I) of the proton of the benzene ring with the peak shifted from 7.2–7.6 ppm (Ix) to 6.6–7.1 ppm (Iy). The conversion to droplet material (%) was equated to 100×Iy/(Ix+Iy) %. Data are presented as mean values +/- SD. The number of samples is 3. Source Data is provided in the Source Data File.

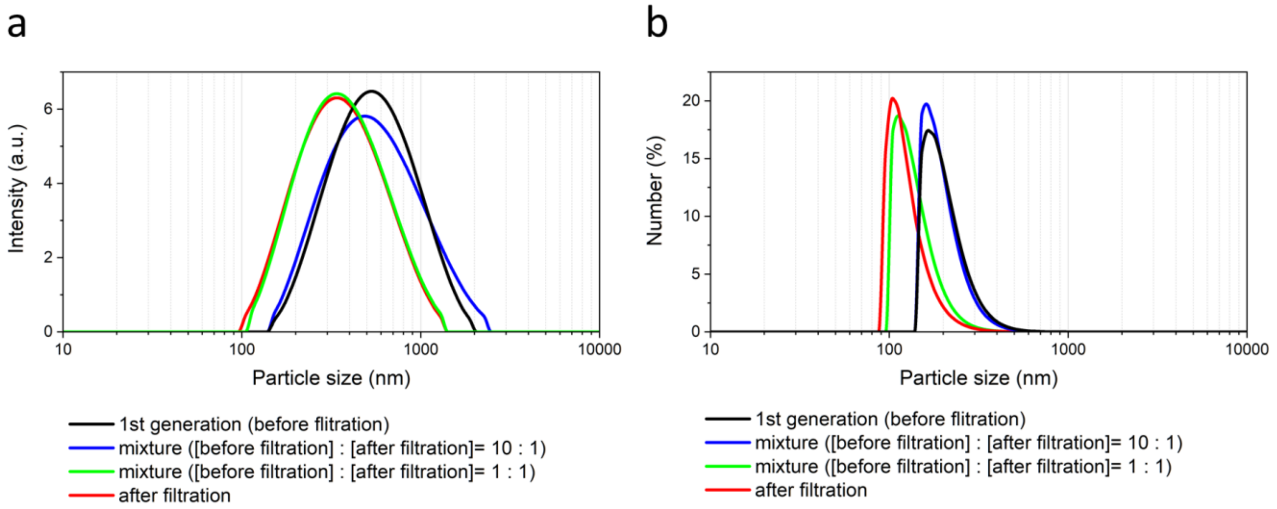

Fig. 15 a Scattering intensity measurement of the mixture before/after extruded droplet dispersion. **b** Size measurements of the mixture before/after extruded droplet dispersion. Mixed droplet dispersions (before filtration:after filtration =1:1 or 10:1 mol%) before and after extrusion through a 100-nm mesh polycarbonate filter were measured with a dynamic light-scattering, particle-size-distribution analyser. The distribution of the scattering intensity of the mixed solution was intermediate between before-filtration solution and after-filtration solution, and at least both distributions were detected. In contrast, most of the droplets in the particle size distribution were ~100 nm in diameter. The implication is that the number of droplets was much higher in the extruded dispersion than in the unextruded solutions with the same amino acid concentration. In other words, these results strongly suggest that if a monomer precursor with the same amino acid concentration is added to a droplet dispersion, and if the

chemical reaction that then proceeds outside the droplet generates new, smaller droplets, a new distribution is produced, and the original distribution disappears. The experimental results in Figure 4b show that neither of these events occurred. The conclusion is that the chemical reaction proceeded almost entirely inside the droplets.

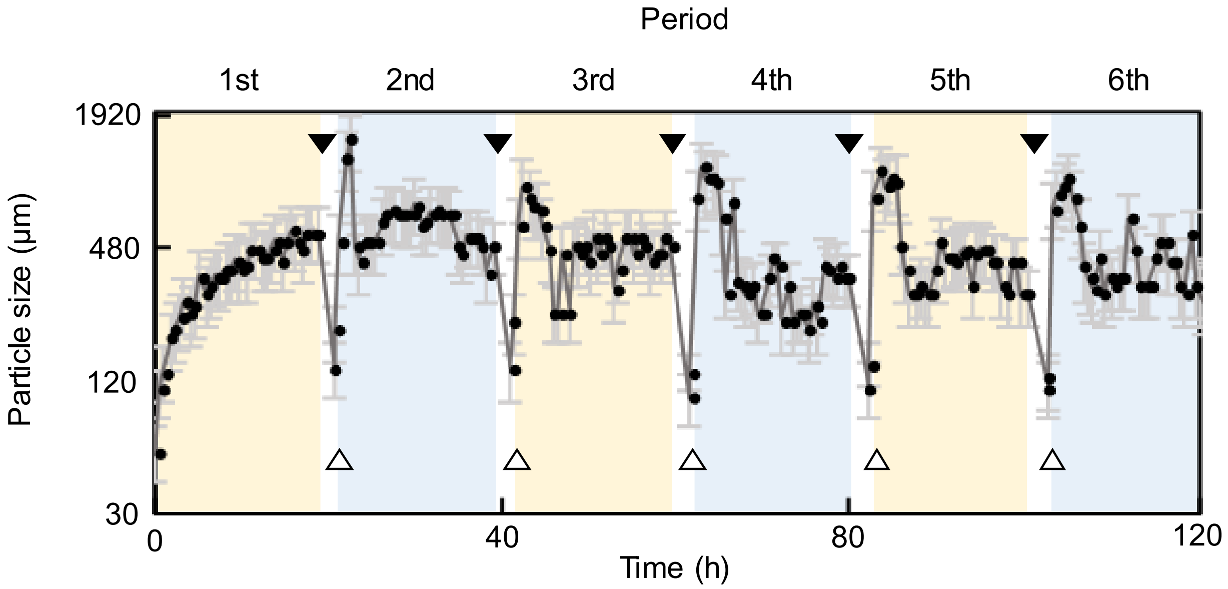

Fig. 16 Raw image of Figure 5b. Source Data is provided in the Source Data File.

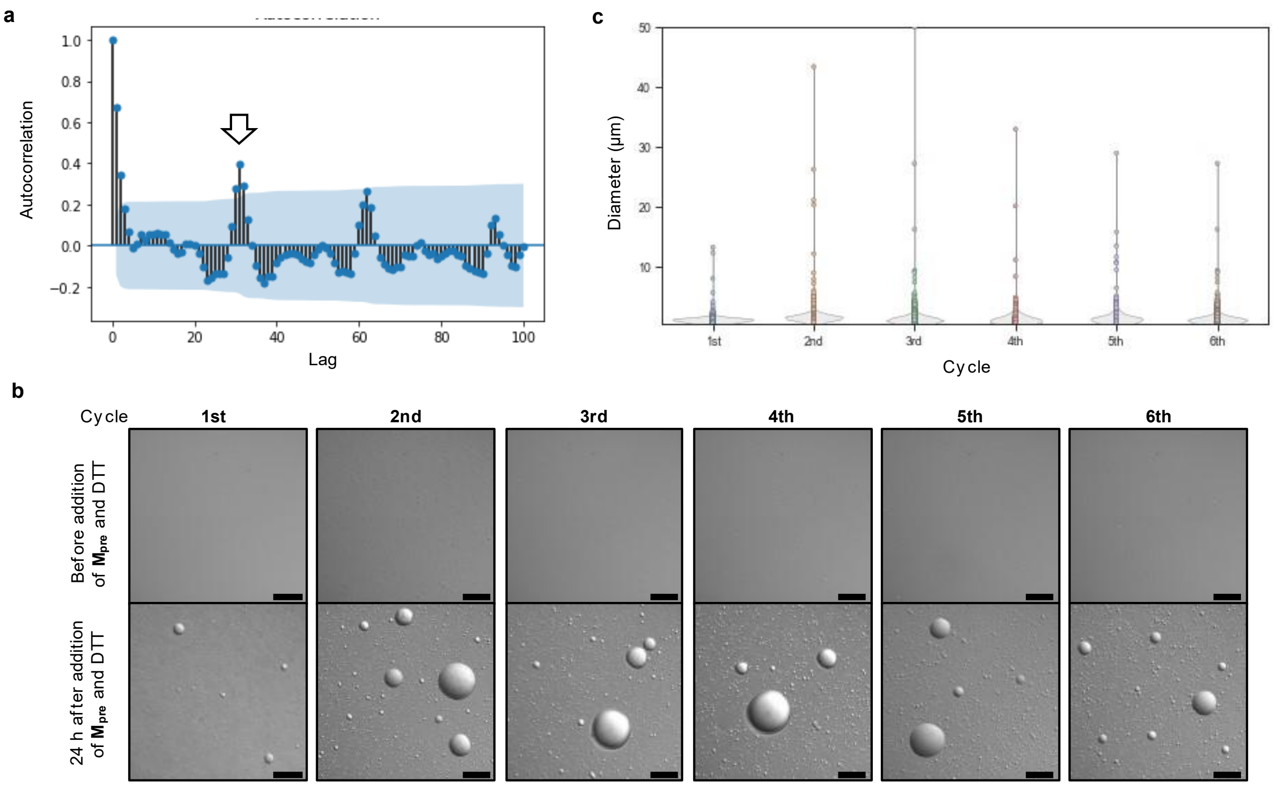

Fig. 17 Changes in LLPS droplet size during the growth–division process induced by repeated nutrient addition and stimulus cycles. **a** Correlogram of the LLPS droplet size changes with time shown in Figure 5b. No significant differences emerged between the size distributions of the droplets measured in each cycle. The lag is the number of measurement points that were shifted in calculating the autocorrelation coefficient. (If lag = 0, there was no shift, and the autocorrelation coefficient was 1.) Because the number of measurement points per period was 33 in this study, the significant maximum correlation coefficient, which exceeded the 95% confidence interval at lag 33 for the first time, implied a high particle-size recursiveness for each cycle. **b** DIC microscopy images before and 20 h after addition of $\mathbf{M}_{\mathbf{pre}}$ and DTT in each cycle. Sale bars represent 50 μm. **c** Particle size distributions obtained from the analysis of DIC microscopy images 20 h after addition of nutrient in each cycle. The result was verified by five trials. Source Data of 17a and 17c is provided in the Source Data File.

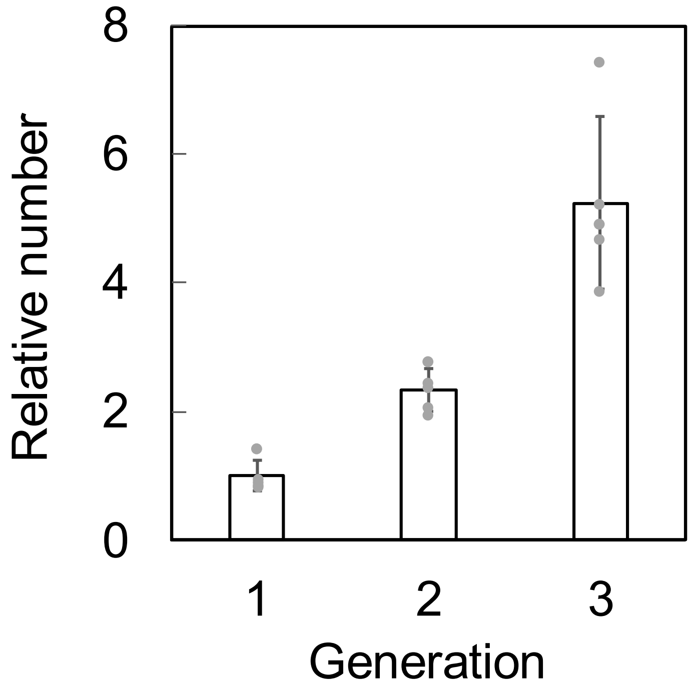

Fig. 18 Relative number of droplets after each generation. Each dispersion was obtained with the same protocol as in Figure 5b and was observed under a phase-contrast microscope. Droplets with sizes >1 μm in the microscope images (290 μm × 217 μm), which appeared after addition of the precursor $\mathbf{M_{pre}}$, were counted (number of droplets in a field, $N > 1000$). The figure shows the number of droplets after each generation relative to the number after the first generation. Corresponding data were overlayed as dot plots. Data are presented as mean values +/- SD. The number of samples is 5. Source Data is provided in the Source Data File.

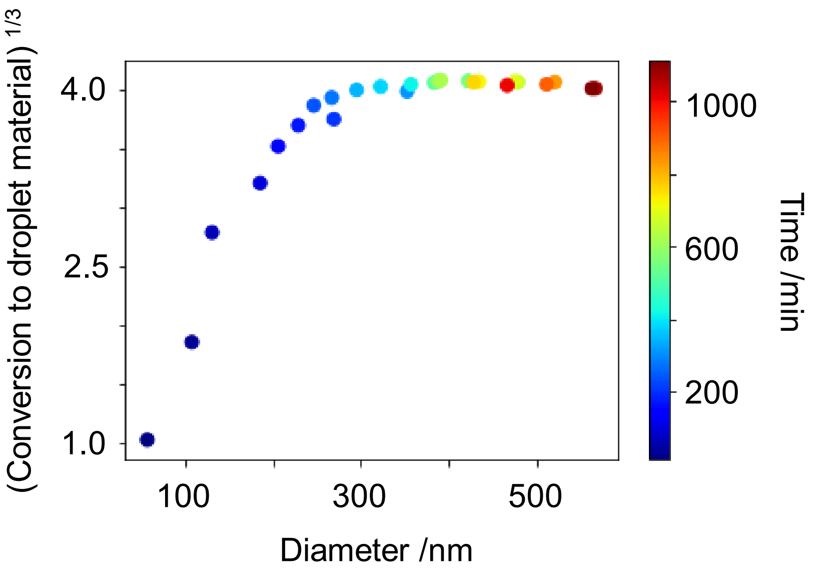

Fig. 19 Time-course changes in droplet size and cube root of conversion to droplet material. Change in particle size and reaction rate over time. The cube root of the conversion to droplet material was calculated from Figure 3c. The reaction rate reached a plateau at about 3 h. Although the droplet sizes continued to increase thereafter, the rate of increase of the particle sizes was significantly lower than the rate before 3 h. Source Data is provided in the Source Data File.

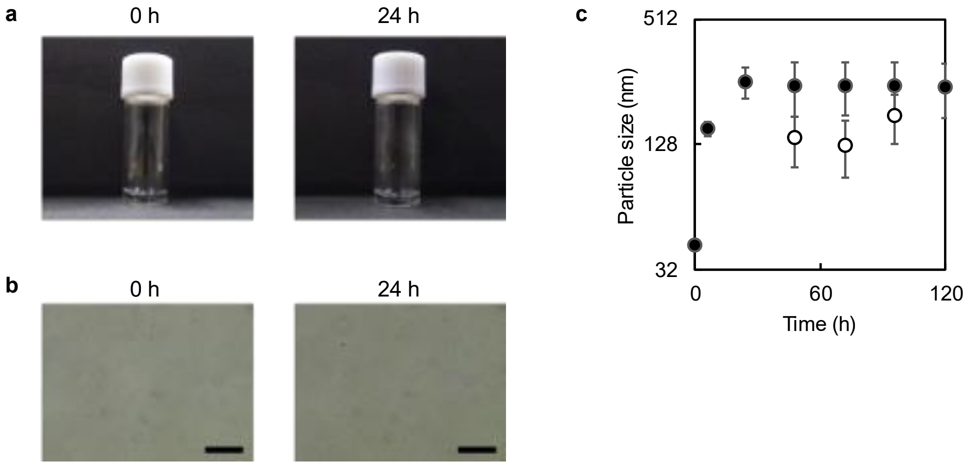

Fig. 20 **a** Comparison of extruded droplet dispersion at 0 and 24 h after addition of water. **b** Comparison of phase contrast microscopy images of droplet dispersion at 0 and 24 h. Scale bars represent 50 µm. To show that the increase in size of the droplets was not due to fusion of droplets, we performed an experiment with only droplets. During that experiment, the droplets did not increase in

size. A droplet dispersion was prepared 24 h after the addition of $\mathbf{M_{pre}}$ (5 mg) and DTT (4 mg) to water (1 mL). The droplet dispersion was filtered through a 100-nm pore size membrane filter, and the solutions were compared immediately and 24 h after the addition of an equal amount of water. The result was verified by five trials. **c** Change in droplet size of droplet dispersion with added $\mathbf{M_{pre}}$ (●) and filtered droplet dispersion 24 h after $\mathbf{M_{pre}}$ addition (○). When the droplet solution was not subjected to filtration, a droplet size increased up to 24 h after the $\mathbf{M_{pre}}$ addition. The size had not changed after 24 h (at least for 120 h after the addition). On the other hand, when the solution was filtrated 24 h after $\mathbf{M_{pre}}$ addition, the particle size decreased to around 100 nm immediately after filtration, and thereafter the particle size remained almost constant at least until 96 h after addition. However, it was not possible to measure the droplet size of the filtrated dispersion 120 h after $\mathbf{M_{pre}}$ addition. This is because the loss of droplets due to the volatilization of BnSH, which is a droplet component, becomes not negligible. These results strongly suggested that the droplet size after the second generation was increased by self-reproduction of droplets through $\mathbf{M_{pre}}$ addition. Data are presented as mean values +/- SD. The number of samples is 3. Source Data is provided in the Source Data File.

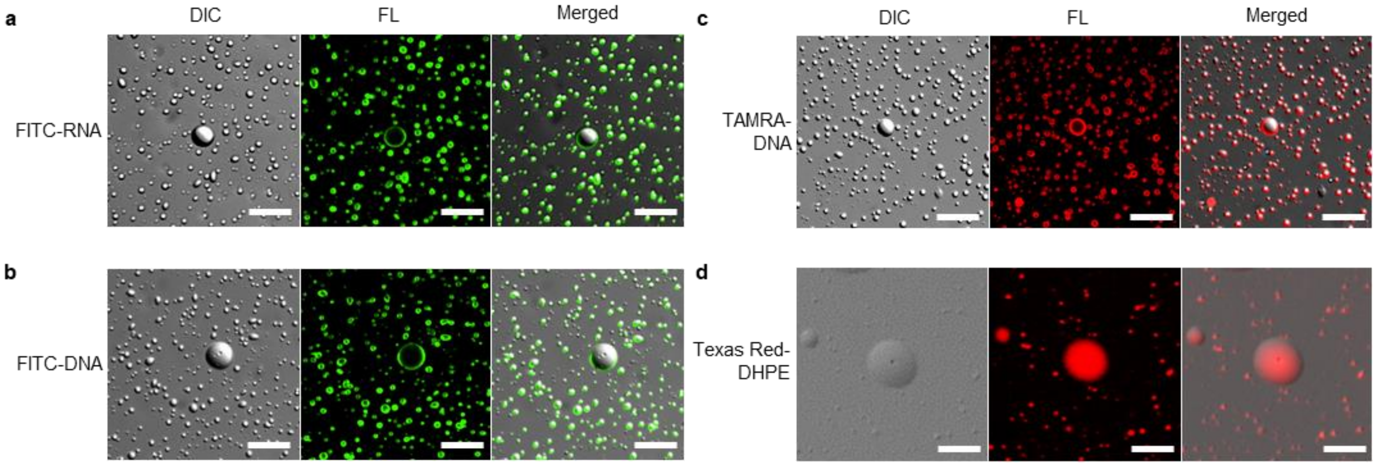

Fig. 21 CLS fluorescence microscopy images of the dispersion of LLPS droplets after addition of solutions of **a** FITC-RNA, **b** FITC-DNA, **c** TAMRA-DNA, or **d** Texas Red-DHPE; both the individual (FL) and merged signals are shown. The concentrations of the reagents in all solutions were 1 μM. The

LLPS droplet dispersions were captured at 30 min after addition of nucleic acid solutions (**a**–**c**) and at 16 h after addition of the lipid solution (**d**). The results were verified by five trials. The scale bars represent 20 μm.

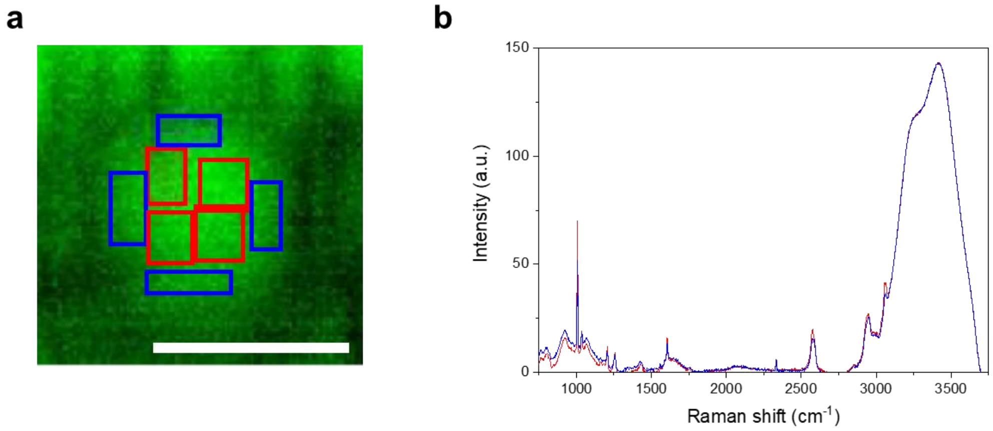

Fig. 22 Raman microspectrometry of an LLPS droplet. **a** Raman imaging of an LLPS droplet. The wavelength of the laser used in the microscope was 532 nm. The red rectangles represent the centre of the droplet. The blue rectangles represent the periphery of the droplet. The scale bar represents 20 μm. For each region (4 red regions and 4 blue regions), measurements were performed twice. **b** Raman spectra of the centre and periphery of a measured droplet. Red line is the spectrum of the centre of the droplet. The average spectrum was calculated from the spectra obtained for each of the four rectangular regions outlined in red in Supplementary Figure 22a. The average spectrum of the centre of the droplet and the baseline-corrected spectrum are shown. Based on the obtained spectral data, C(Bn/water) was calculated to be 0.49 ± 0.044. The blue line represents the periphery of the droplet. The average spectrum obtained for the four regions outlined in blue is shown in Supplementary Figure 22. P(Bn/water) was calculated to be 0.36 ± 0.094. The p-value between C(Bn/water) and P(Bn/water) was 0.021 ($p < 0.05$) and was calculated based on a Mann-Whitney U test (two-sided). The p of 0.021 was considered statistically significant. Adjustments were not made for multiple comparisons. Source Data is provided in the Source Data File. This result indicates that the centre of the droplets was more hydrophobic than the periphery.

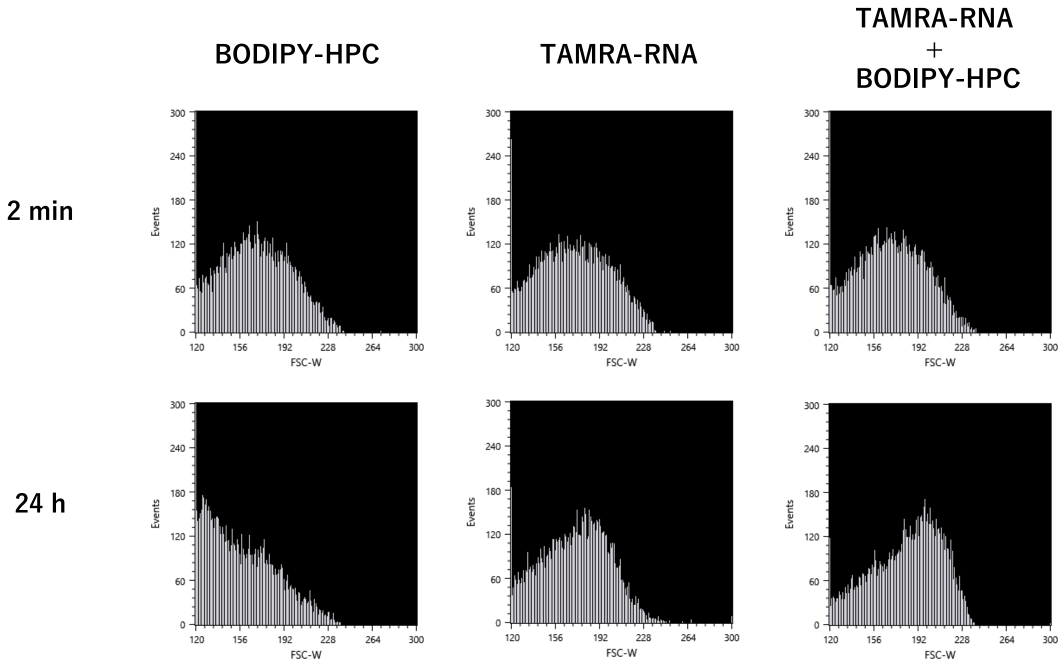

Fig. 23 FACS analysis of particle size distribution of the droplets at 2 min (upper) or 24 h (bottom) after addition of BODIPY-HPC and/or TAMRA-RNA. In these histograms, the x-axis represents the forward-scattering light pulse width (FSC-W), which reflects the particle size, and the y-axis represents the number of events. When BODIPY-HPC was added to the LLPS dispersion, the distribution shifted significantly to the left in the histogram after 24 h compared to the distribution after 2 min. This means that the particle size of LLPS droplets decreased. In contrast, no such decrease in FSC-W values, that is, a decrease in the droplet size, was found when only TAMRA-RNA or both TAMRA-RNA and BODIPY-HPC were added to the droplet. These results indicate that TAMRA-RNA suppressed the size-reduction effect due to addition of BODIPY-HPC.

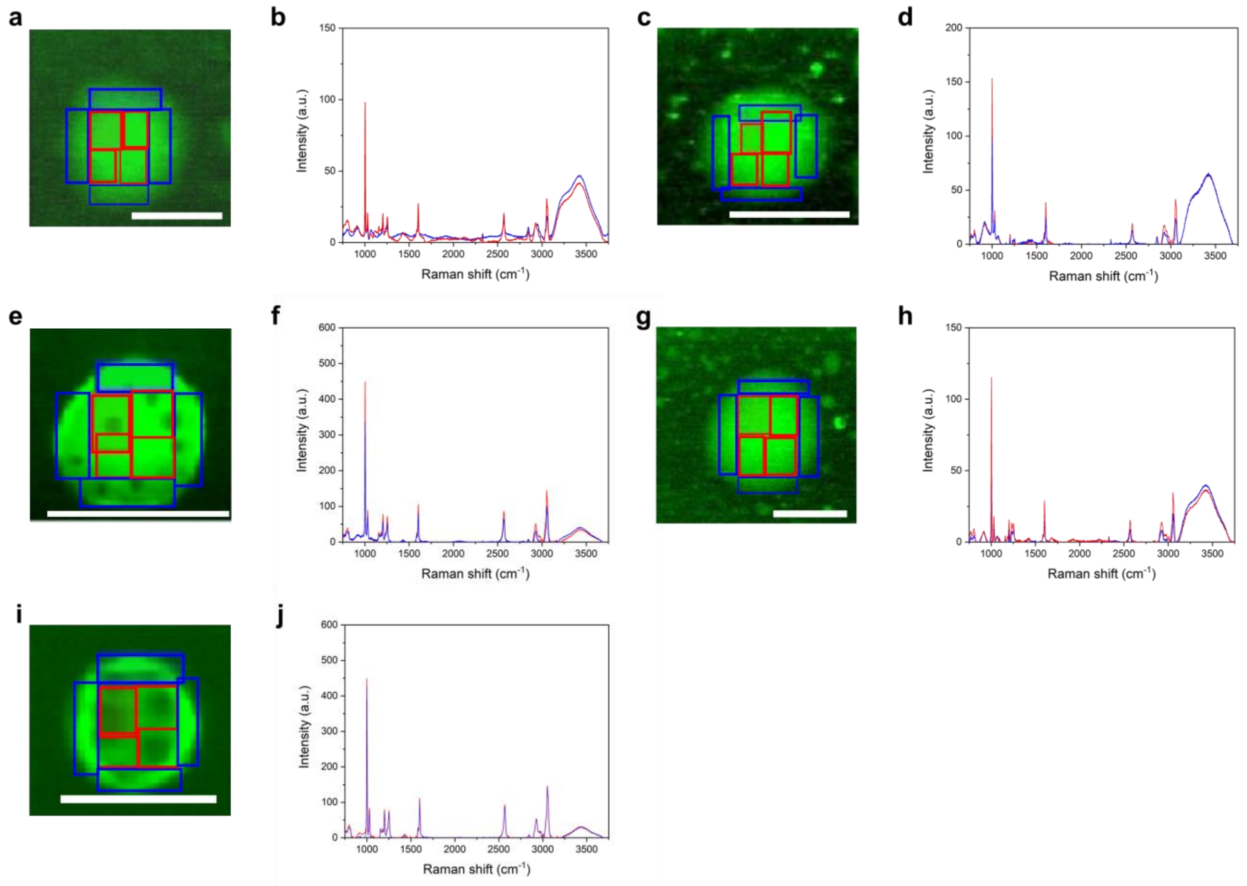

Fig. 24 Raman microspectroscopic images of LLPS droplets after incorporation of RNA or DNA and/or lipids. RNA, DNA, and/or lipids were added to the droplets to examine the ability of the droplets to incorporate and retain nucleic acids/lipids. Raman microspectroscopy was conducted using RNA or DNA as the nucleic acid and DPPC with double-stranded saturated alkyl chains as a lipid. Raman images (**a, c**, **e**, **g**, **i**) were obtained using a laser with a wavelength of 532 nm for 10 s; scale bars represent 20 µm. For each region (4 red regions and 4 blue regions), measurements were performed twice. In the obtained spectra (**b, d**, **f**, **h**, **j**), each p-value between C(Bn/water) and P(Bn/water) was provided by Mann-Whitney U test (two-sided) provided and adjustments were not made for multiple comparisons. **a**, **b** Raman imaging and spectra of an LLPS droplet containing RNA. In the image, the red rectangles represent the measured central parts of the droplet and the blue rectangles represent the measured peripheral parts of the droplet. In the Raman spectra, the red line represents the average spectrum for the centre of the droplet calculated from the spectra obtained for each of the four red

rectangles; the baseline-corrected spectrum is shown. Based on the obtained spectral data, C(Bn/water) was calculated to be 2.4 ± 0.26. The blue line represents the average spectrum for the periphery of the droplet. The P(Bn/water) was calculated to be 1.2 ± 0.28. A Mann-Whitney U test provided a *p*-value between C(Bn/water) and P(Bn/water) of 0.049, which was considered statistically significant ($p < 0.05$). This result indicated that the centre of the droplet was more hydrophobic than the periphery. The intensity of the RNA's phosphate peaks (784 cm^{-1}, 830 cm^{-1}, 1090 cm^{-1})[2] in the spectra were quite weak. The possibility that this weakness was due to intrusion of some RNA into the droplet is quite small. The spectra more likely reflect the constituents of the droplet and/or the possibility that the RNA was damaged by the laser irradiation at the time of this measurement. When RNA was added, the central region was also found to be more hydrophobic. Supplementary Table 1 summarizes these results. **c**, **d** Raman imaging and spectra of an LLPS droplet containing DNA. Based on the obtained spectral data, C(Bn/water) was calculated to be 3.2 ± 0.071 and P(B/W) was calculated to be 1.8 ± 0.28. The *p*-value between C(Bn/water) and P(Bn/water) was 0.12 ($p > 0.05$). Similar to the RNA findings, the intensity of the peaks for the DNA were also weak. **e**, **f** Raman imaging and spectra of an LLPS droplet containing DPPC. Raman imaging revealed internal hydrophobic droplets that were clearly visible but were not apparent in samples without lipids (Supplementary Figures 22a, 24a, c). In the spectra, peaks were found in the DPPC-derived region (1050–1200 cm^{-1})[2]. Based on the obtained spectral data, C(Bn/water) was calculated to be 11 ± 1.2 and P(Bn/water) was calculated to be 7.9 ± 0.85. The *p*-value between C(B/W) and P(Bn/water) was 0.033 ($p < 0.05$). **g**, **h** Raman imaging and spectra of an LLPS droplet containing RNA and DPPC. Based on the obtained spectral data, C(Bn/water) was calculated to be 3.2 ± 0.085 and P(Bn/water) was calculated to be 1.9 ± 0.055. The *p*-value between C(Bn/water) and P(Bn/water) was 0.12 ($p > 0.05$). **i**, **j** Raman imaging and spectra of an LLPS droplet containing DNA and DPPC. Based on the obtained spectral data, C(Bn/water) was calculated to be 12± 1.3 and P(Bn/water) was calculated to be 10 ± 0.66. The *p*-value between C(Bn/water) and P(Bn/water) was 0.034 ($p < 0.05$). Source Data of 24b, 24d, 24f, 24h and 24j is

provided in the Source Data File. A comparison of Supplementary Figures 22 and 24 revealed that the intensity of the peaks derived from water (3400 cm^{-1}) decreased, and the Bn/water values in both the centre and periphery of the droplets increased. This suggests that the water inside the droplet might be replaced by hydrophilic DNA and amphiphilic phospholipids.

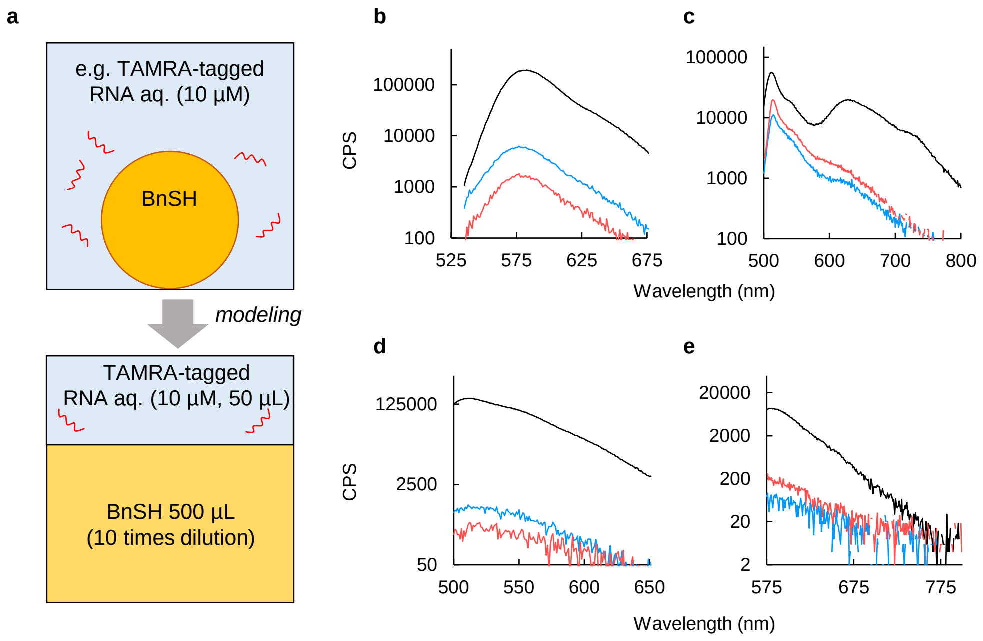

Fig. 25 Fluorescence spectroscopic analysis of the concentrations of oligopeptides in biopolymers. **a** Schematic of this experiment. In the case of addition of biopolymers to a droplet, i.e., the nucleic acids and lipids added with BnSH, we investigated the extent to which those biopolymers were incorporated into BnSH and water. In a typical experiment, each solution containing a fluorescently labelled biopolymer (nucleic acid /lipid, 10 µM, 50 µL) was layered onto a 10-times diluted BnSH solution (500 µL). The fluorescence intensities of the biopolymers in the water and BnSH solution were measured with a fluorescence spectrometer. **b**–**e** Fluorescence spectra of aqueous solutions containing RNA oligomers or phospholipids loaded onto the BnSH-layer solutions: black, before loading; red, aqueous solution containing oligopeptides 24 h after loading; blue, aqueous solution without oligopeptides 24 h after loading. **b** TAMRA-RNA. **c** BODIPY-HPC. **d** FITC-RNA. **e** Texas Red-DHPE. In the presence of RNA, the fluorescence intensity of the aqueous layer containing the oligopeptides was less than one-third that of the aqueous layer without the oligopeptides, and it decreased to 1% or

less of intensity before loading (**b**, **d**). However, in the case of phospholipids, no differences in fluorescence intensities associated with the presence of the oligopeptides were observed, although the fluorescence intensity decreased to nearly one-third of that before layering (**c**, **e**).

Supplementary Table

Table 1. Summary of Raman microspectroscopic analysis of an LLPS droplet with/without RNA or DNA and/or lipids.

Supplementary Figure	RNA	DNA	Phospholipid	C(Bn/water)	P(Bn/water)	*p* value (two-sided)
22b	–	–	–	0.49 ± 0.044	0.36 ± 0.094	0.021
24b	+	–	–	2.4 ± 0.26	1.2 ± 0.28	0.049
24d	–	+	–	3.2 ± 0.071	1.8 ± 0.28	0.12
24f	–	–	+	11 ± 1.2	7.9 ± 0.85	0.033
24h	+	–	+	3.2 ± 0.085	1.9 ± 0.055	0.12
24j	–	+	+	12 ± 1.3	10 ± 0.66	0.034

Supplementary Note

Note 1. Plausible prebiotic synthesis of components. The temperature of monomer synthesis and polymerisation in this system was below 100°C, and the pressure was 1 atm. The pH of monomer synthesis was ~7 and the pH of polymerisation was 3–11 (Supplementary Figure 7). Water is a liquid under these conditions, which include environmental conditions found on primitive Earth, although they do not include conditions found in hydrothermal vents, such as the conditions found among the rocks in the Isua region of southwestern Greenland[3]. For example, in and around a geyser, the combinations of temperature and pH that allow for dilution and shearing by the inflow of water could be achieved.

The monomers in this study were thioesters, which can be formed on the surface of iron-containing minerals, and this system was a non-prebiotic model based on thioesters[4]. It is true that benzyl

mercaptan, which is a leaving group, would have been difficult to synthesize in the environment of primitive Earth and was thus regarded as a model of a primitive hydrophobic thiol. However, it would have been possible to form benzyl mercaptan from primitive molecules in a stepwise manner[5,6] by changing the organic solvent, and, in the process, producing conditions not very different from the pre-biological environment. Our system can thus be regarded as a model for the origin of life because we used model molecules as leaving groups and achieved growth by self-production in a prebiotic environment.

In addition, the nucleic acids and lipids used in this study were used on the assumption that they could have originated in a prebiotic environment. Nucleic acids and lipids might have been generated in a prebiotic environment by the following reaction pathways. Vigorous formation of nucleic acids (RNA/DNA) in a primitive environment has been reported by Sutherland et al.[7] in a paper that we cited. The formation of fatty acids such as formic acid, acetic acid, and pyruvic acid from carbon dioxide and subsequently the formation of phospholipids on the surface of several minerals has been verified in a CO_2-reduction system[8]. However, those studies have not ruled out the possibility that nucleic acids and lipids were gradually synthesised inside proliferating peptide droplets in a prebiotic environment by provision of a hydrophobic field in water.

Note 2. Separation of droplets into water phase and oil phase by centrifugation.

The total volume of the droplets in the sample was too small (ca. 1 μL/ sample) to be centrifuged and analysed by HPLC.

Supplementary Movie

Movie 1. Droplet formation. This differential interference microscopy video shows the formation of an LLPS droplet after the addition of $\mathbf{M_{pre}}$ and DTT. The movie covers the time from immediately after addition of $\mathbf{M_{pre}}$ and DTT to 12 h later. The playback speed is 4320×. The scale bar represents 50 μm.

Movie 2. Droplet fusion. This differential interference microscopy video shows droplet fusion during formation of droplets (trimmed version of Supplementary Movie 1). The movie was recorded from immediately after addition of $\mathbf{M_{pre}}$ and DTT to 12 h later. The playback speed is 4320×. The size of the field of view is 50 µm × 50 µm.

Supplementary References

1. Matsuo, M. *et al.* DNA length-dependent division of a giant vesicle-based model protocell. *Scientific Reports* **9**, doi:10.1038/s41598-019-43367-4 (2019).
2. Kuhar, N., Sil, S., Verma, T. & Umapathy, S. Challenges in application of Raman spectroscopy to biology and materials. *RSC Advances* **8,** 25888-25908, doi:10.1039/C8RA04491K (2018).
3. Pons, M.L. *et al*., Early Archean serpentine mud volcanoes at Isua, Greenland, as a niche for early life, *Proceedings of the National Academy of Sciences* **108** (43), 17639-17643 (2011).
4. Camprubi E., Jordan S. F., Vasiliadou R. Lane N. Iron catalysis at the origin of life. *IUBMB Life* **69**, 373-381, doi:10.1002/iub.1632 (2017).
5. Zhou, Minghe, China, CN106397120 A 2017-02-15.
6. Liu, Xiaozhi; *et al*, China, CN101186591 A 2008-05-28.
7. Xu, J., Chmela, V., Green, N. *et al.* Selective prebiotic formation of RNA pyrimidine and DNA purine nucleosides. *Nature* **582**, 60-66 (2020).
8. Preiner, M., Igarashi, K., Muchowska, K.B. *et al.* A hydrogen-dependent geochemical analogue of primordial carbon and energy metabolism. *Nat. Ecol. Evol.* **4**, 534-542 (2020).
