# Supplementary material for: Proliferating coacervate droplets as the missing link between chemistry and biology in the origins of life
Source: Nat Commun. 2021 Sep 24;12:5487. doi: 10.1038/s41467-021-25530-6 (PMC8463549; doi:10.1038/s41467-021-25530-6)
Supplement: Supplementary file 3 — Description of Additional Supplementary Files [file 41467_2021_25530_MOESM3_ESM.docx]

Description of Additional Supplementary Files

Title: Supplementary Movie 1.

Description: Droplet formation. This differential interference microscopy video shows the formation of an LLPS droplet after the addition of Mpre and DTT. The movie covers the time from immediately after addition of Mpre and DTT to 12 h later. The playback speed is 4320×. The scale bar represents 50 μm.

Title: Supplementary Movie 2.

Description: Droplet fusion. This differential interference microscopy video shows droplet fusion during formation of droplets (trimmed version of Movie S1). The movie was recorded from immediately after addition of Mpre and DTT to 12 h later. The playback speed is 4320×. The size of the field of view is 50 μm × 50 μm.
